# Potential protective role of *Bifidobacteria* in myopia prevention: evidence from full-length 16S rRNA sequencing and bidirectional Mendelian randomization analysis

**DOI:** 10.3389/fmed.2025.1634120

**Published:** 2025-08-13

**Authors:** Guodong Tang, Yibo Han, Xiaoqi Gong, Xuejing Wang, Jing Li, Jun Zhang, Junru Wang, Jike Song, Hongsheng Bi

**Affiliations:** ^1^Ophthalmology and Optometry Department, Affiliated Eye Hospital of Shandong University of Traditional Chinese Medicine, Jinan, China; ^2^Medical College of Optometry and Ophthalmology, Shandong University of Traditional Chinese Medicine, Jinan, China; ^3^Shandong Key Laboratory of Integrated Traditional Chinese and Western Medicine for Prevention and Therapy of Ocular Diseases, Jinan, China

**Keywords:** myopia, gut microbiota, Mendelian randomization, full-length 16S rRNA gene sequencing, Bifidobacterium

## Abstract

**Introduction:**

The increasing prevalence of myopia worldwide poses significant public health concerns. Accumulating evidence suggests a potential link between ocular diseases and the gut microbiota (GM); however, whether the GM directly contributes to myopia development remains to be established.

**Methods:**

This study investigated the potential causal link between the GM and myopia through bidirectional Mendelian randomization (MR) analysis, further validated by experiments conducted on a form-deprivation myopia (FDM) guinea pig model. Bidirectional two-sample MR analysis was performed using genome-wide association study summary statistics comprising data on 196 GM taxa from the MiBioGen consortium and myopiaassociated data from the FinnGen database. Instrumental variables were carefully selected according to predetermined standards. Subsequently, MR estimates were calculated using the inverse variance weighted, MR-Egger regression, and weighted median approaches, along with supplementary sensitivity evaluations. Concurrently, FDM was experimentally induced in guinea pigs, and fecal samples were subjected to comprehensive full-length 16S rRNA gene sequencing analysis.

**Results:**

MR analysis identified five bacterial taxa linked to the risk of myopia. Specifically, higher Bifidobacterium abundance was associated with lower myopia risk (odds ratio = 0.834, 95% confidence interval = 0.705–0.986, *p* < 0.05). Animal experiments validated the MR findings, demonstrating a significant enrichment of Bifidobacteria in control animals.

**Discussion:**

Conclusively, supplementation with Bifidobacteria is a potential strategy for reducing the risk of myopia. Future research should focus on developing and testing Bifidobacterium-based interventions to validate their effectiveness in controlling myopia.

## 1 Introduction

Estimates suggest that almost half of the world’s population will develop myopia by 2050, imposing a significant burden on the global healthcare systems (1). Myopia development is influenced by both hereditary and environmental influences (2). For instance, prolonged near work (especially at short viewing distances) and higher levels of education are associated with an increased risk of myopia, whereas spending more time outdoors tends to be protective (3). Notably, high myopia substantially increases the likelihood of vision-threatening eye disorders such as retinal detachment, glaucoma, and cataract formation (4, 5). Uncorrected refractive errors and pathological myopia are major contributors to visual impairment and, in some cases, blindness (6, 7). Therefore, preventing or delaying the onset of myopia is crucial.

Recent research has revealed a potential connection between myopia and gut microbiota (GM). The GM constitutes a complex ecosystem comprising approximately 4 × 10^13^ symbiotic organisms, including bacteria, protozoa, fungi, archaea, and viruses (8, 9). Over the past decade, mounting evidence has identified gut microbial dysbiosis as a crucial factor underlying the initiation and progression of numerous diseases (10). Recent findings indicate GM involvement in various ocular pathologies, including optic neuritis (11), age-related macular degeneration (12), and diabetic retinopathy (13). Nevertheless, the relationship between GM and myopia remains incompletely characterized and warrants further investigation. Li et al. (14) demonstrated significant alterations in GM and metabolite profiles in myopic mice, with dysbiosis and plasma metabolomic changes suggesting gut barrier disruption. Subsequent investigations have explored GM-associated metabolic pathways; Wu et al. (15) characterized *Ruminococcus albus* and glutamate metabolic pathways as critical factors in form-deprivation myopia (FDM). In a cohort analysis of 52 adults, Omar et al. (16) observed that myopic participants displayed increased abundances of multiple gut bacterial genera, including *Bacteroides*, *Faecalibacterium*, *Dorea*, *Roseburia*, and *Blautia*, relative to non-myopic controls. While these investigations have identified associations, their limited sample sizes and absence of causal inference preclude establishing a definitive causal relationship between GM and myopia.

Mendelian randomization (MR) approaches have gained prominence in recent years for identifying causal risk factors underlying myopia (17–19). Notably, Mountjoy et al. employed bidirectional MR analysis to establish that extended educational duration contributes to increasing myopia prevalence (19). Additionally, bidirectional MR revealed a strong bidirectional genetic causal relationship between myopia and primary open-angle glaucoma, primarily mediated through intraocular pressure (17). MR leverages genetic variations as “natural randomized experiments” to replicate the effects of randomized controlled trials. This approach provides a robust framework for evaluating the causal relationships between modifiable exposures or risk factors and clinically relevant outcomes (20). Through application of Mendelian segregation and independent assortment principles, MR minimizes confounding biases that frequently compromise traditional observational epidemiology, enabling more precise delineation of causal pathways in complex phenotypic traits (21).

This investigation employed bidirectional two-sample MR analysis to comprehensively evaluate potential causal relationships between diverse GM taxa and myopia. To explore whether myopia might reciprocally influence GM composition, we performed reverse MR analysis. For validation of MR findings, we conducted full-length 16S rRNA gene sequencing on fecal samples from form-deprived myopic guinea pigs using third-generation sequencing technology. Full-length 16S rRNA gene sequencing provides enhanced species-level taxonomic resolution compared to traditional short-read 16S approaches (22). These advanced techniques enable the accurate identification of microbial taxa at the species and strain levels, facilitating the precise characterization of myopia-associated microbiota structures. This investigation, to our knowledge, represents the first integration of bidirectional MR with full-length 16S rRNA sequencing to elucidate the gut microbial taxa contributing to the onset and progression of myopia. Our findings provide a foundational framework for future investigations exploring gut microbiota modulation as a therapeutic strategy for myopia management.

## 2 Materials and methods

### 2.1 Study design

**Figure 1:**
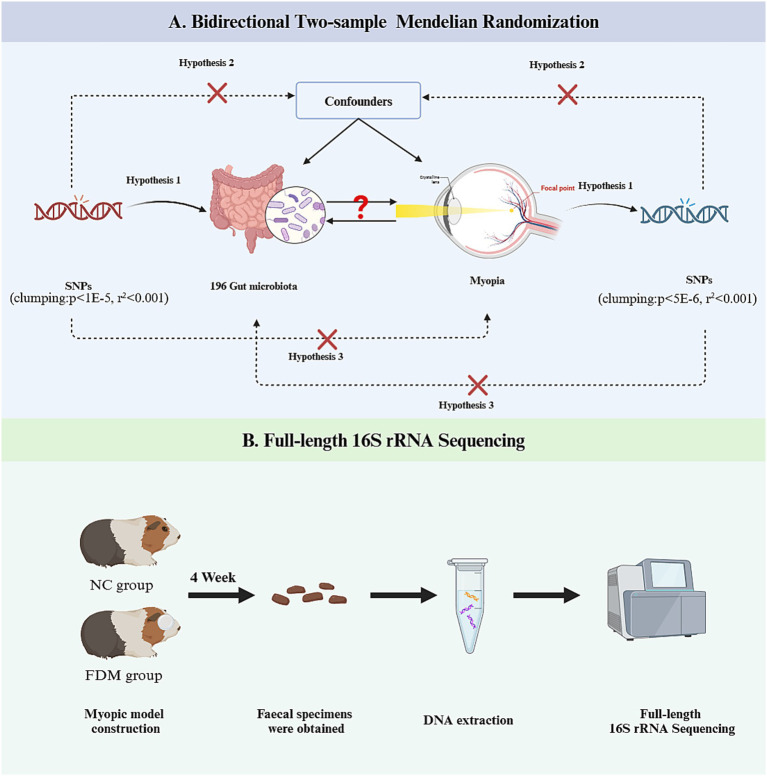
Overview of the research workflow. FDM, form-deprivation myopia; NC, normal control; SNPs, single nucleotide polymorphisms. **(A)** Illustrates the analytical workflow of Mendelian randomization; **(B)** depicts the experimental workflow of 16S rRNA sequencing.

Using a two-sample bidirectional MR framework, this study examined potential causal relationships between GM and myopia onset by leveraging summary-level data from an independent genome-wide association study. For the MR methodology to yield valid inferences, it was necessary to adhere to three foundational assumptions: (1) the chosen genetic instruments must demonstrate robust statistical associations with the exposure variable; (2) these instrumental variants should exert effects on the outcome solely through their influence on the exposure of interest; and (3) genetic proxies must remain unassociated with confounding variables that could otherwise distort the observed exposure-outcome association. This analytical approach ensures a rigorous evaluation of causality while minimizing bias from pleiotropic pathways or residual confounding (23). Guinea pigs were selected to validate the *in vivo* experiments for MR analysis (Figure 1).

### 2.2 Data sources

GM data encompassing 196 taxa were obtained from the MiBioGen consortium, which performed meta-analysis on data of 18,340 individuals (24). Myopia outcome data were acquired from the Finnish FinnGen Biobank. FinnGen identified myopia cases using ICD-10 code H52.1 (nearsightedness) within health registries (https://r9.risteys.finngen.fi/endpoints/H7_MYOPIA). The FinnGen dataset comprised 4,106 myopia cases and 394,028 controls of European ancestry with over 1.0 million genotyped SNPs. All database populations consisted of individuals of European descent. Data were fully anonymized, with no individual-level information accessible for this investigation.

### 2.3 IVs selection

SNPs associated with the GM were selected with a combined threshold (*p* < 1 × 10^−5^) and linkage disequilibrium analysis (PLINK v1.9) with a distance >10,000 kb and r^2^ < 0.001, ensuring the robustness of Instrumental variables (IVs) validated by F-statistic >10. For myopia-associated IVs in the reverse MR analysis, a stricter threshold (*p* < 5 × 10^−6^) was applied (Supplementary Tables S1, S2).

### 2.4 MR analysis and sensitivity analysis

The inverse variance weighted (IVW) method served as our primary analytical approach for evaluating potential associations between GM and myopia. We performed additional analyses using MR-Egger regression and weighted median models to assess the robustness of causation estimates. Consistent directional results across different MR analytical models strengthened the credibility of causal inferences. We employed the MR-Egger intercept test to detect horizontal pleiotropy (25) and utilized MR-PRESSO to identify outliers and adjust for horizontal pleiotropy, with statistical significance set at *p* < 0.05 (26), indicating potential direct instrumental variable effects on outcomes independent of exposure. We applied Cochran’s Q test to assess heterogeneity among genetic variants (27), with *p* < 0.05 indicating significant heterogeneity. SNPs demonstrating high heterogeneity were excluded from analysis. We performed leave-one-out analysis to assess potential bias in MR estimations attributable to individual SNPs (28). Statistical analyses were conducted using R software (version 4.2.1, R Foundation for Statistical Computing, Vienna, Austria) with the two-sample MR package (version 0.5.6) and MR PRESSO package (version 1.0).

### 2.5 Myopia model and groups

We obtained 18 clinically healthy male tricolor guinea pigs from Jinan Jinfeng Laboratory Animal Co. and housed them in a dedicated, ventilated facility under controlled conditions: 12-h light–dark cycles, temperature maintained at 21–25°C, and relative humidity at 40–70%. Animals received food and water *ad libitum*. We randomly allocated animals into FDM and normal control (NC) groups (*n* = 9 each). The FDM model was selected for its reliable induction of significant axial elongation in guinea pigs within short timeframes. Xiao et al. (29) demonstrated no significant differences between FDM and lens-induced myopia models regarding final refraction or axial length, noting guinea pigs’ high sensitivity to FDM. This approach is extensively utilized in myopia research. In the FDM group, we induced myopia by occluding the right eye with translucent latex for 4 weeks, confirming myopia development through increased axial length (AL) and decreased spherical equivalent. NC animals received no experimental intervention. We excluded guinea pigs presenting with ocular disease or pre-existing myopia. Statistical analyses compared treated eyes from FDM animals with eyes from normal control animals, rather than employing paired within-animal comparisons. All procedures received approval from the Ethical Review Committee for Laboratory Animals of Shandong Eye Disease Control Institute (AEHSDUTCM-LAEC2024001) and complied with the ARVO Statement for the Use of Animals in Ophthalmic and Vision Research. Reporting followed ARRIVE guidelines to ensure transparency and completeness.

### 2.6 Refractive status and axial length measurement

We assessed guinea pig refractive errors using a streak retinoscope (YZ24; Six Six Vision Technology Co., Ltd., China). Prior to ocular measurements, we administered one drop of tropicamide/phenylephrine eye medication (Santen Pharmaceutical, Japan) to the conjunctival sac at 5-min intervals, repeating three times. Animals were subsequently placed in darkness for a 30-min adaptation period. An experienced optometrist, operating under dark conditions, determined equivalent refractive errors by averaging vertical and horizontal meridian readings and calculating the mean from three consecutive measurements. We measured axial length using A-scan ultrasound biometry (Cinescan, Quantel Medical, France) following device calibration specifically for guinea pig eyes. Sound-propagation velocities were configured at 1557 m s^−1^ for the anterior chamber, 1723 m s^−1^ for the crystalline lens, and 1,540 m s^−1^ for the vitreous cavity. Prior to each recording, we applied topical anesthesia to the corneal surface using one drop of 0.5% proparacaine hydrochloride (Santen Pharmaceutical, Japan) administered to the conjunctival sac. Final AL values represented the mean of 10 repeated measurements. No animals underwent euthanasia during or following the experiment. All guinea pigs continued under standard husbandry conditions post-experiment, in compliance with ARRIVE guidelines and ethical approval requirements.

### 2.7 DNA extraction, library preparation, and full-length 16S rRNA sequencing

We collected fecal samples from guinea pigs in both NC and FDM cohorts (nine animals per group). Samples underwent immediate snap-freezing and storage at −80°C to preserve molecular integrity. We extracted genomic DNA using a TGuide S96 magnetic-bead soil/feces kit (DP812; Tiangen Biochemical, Beijing, China) and evaluated quality using a femto-pulse capillary system. We amplified the bacterial 16S rRNA locus using universal primers 27F (5′-AGRGTTTGATYNTGGCTCAG-3′) and 1492R (5′-TASGGHTACCTTGTTASGACTT-3′). Polymerase chain reactions (PCRs) were performed in 20 μL volumes containing approximately 10 ng template DNA, 1.5 μL of each primer, 10 μL KOD One™ PCR Master Mix (KMM-101; Bellink Biotechnology, Beijing), and nuclease-free water. We visualized amplicons through agarose gel electrophoresis or LabChip GX Touch analyzer, then purified and converted them into SMRTbell libraries using PacBio Prep Kit v3.0, incorporating damage repair, end polishing, and adapter ligation steps. Libraries underwent cleaning with AMPure PB magnetic beads, quantification on a Qubit fluorometer, and polymerase pairing using the Revio kit. Sequencing was performed for 24 h on a PacBio Revio platform (Annoroad Gene Technology, Beijing, China) following manufacturer’s protocols.

### 2.8 Data analysis of gut microbiome

The effective CCS sequences were processed for clustering and denoising using USEARCH^1^ (30). Taxonomic annotation of the operational taxonomic units (OTU) sequences was performed using the naive Bayesian classifier in the SILVA database^2^ (31). Subsequently, the QIIME2 software^3^ (32) was used to analyze and compare the biodiversity across individual samples and their subgroups. Normalized data were utilized for both alpha and beta diversity analyses. Community richness and evenness were quantified with the Feature, Chao1, Shannon, and Simpson metrics, whereas *β*-diversity dissimilarities were derived from binary Jaccard, Bray–Curtis, and (weighted and unweighted) UniFrac distance matrices. The ordination of the resulting distance space was visualized using principal coordinate analysis and non-metric multidimensional scaling. Differentially abundant taxa were identified using the linear discriminant analysis effect size (LEfSe). Species-level abundance tables were further interrogated using Spearman’s rank correlations, retaining network edges where *ρ* > 0.10 and *p* < 0.05. Finally, functional potentials were inferred using PICRUSt2 against reference databases, such as KEGG and COG.

### 2.9 Statistical analysis

Continuous variables are presented as mean ± standard deviation. Comparisons between two groups were conducted using Student’s t-test or the Mann–Whitney U test. Differences between categorical variables were assessed using the chi-squared test. Spearman’s rank correlation analysis was used to evaluate the relationships between microbial taxa. All statistical analyses were performed using R (version 4.2.1, R Foundation for Statistical Computing) and SPSS (version 26, IBM Corp., Armonk, N.Y., United States).

## 3 Results

### 3.1 Effect of genetically determined GM on myopia

**Figure 2:**
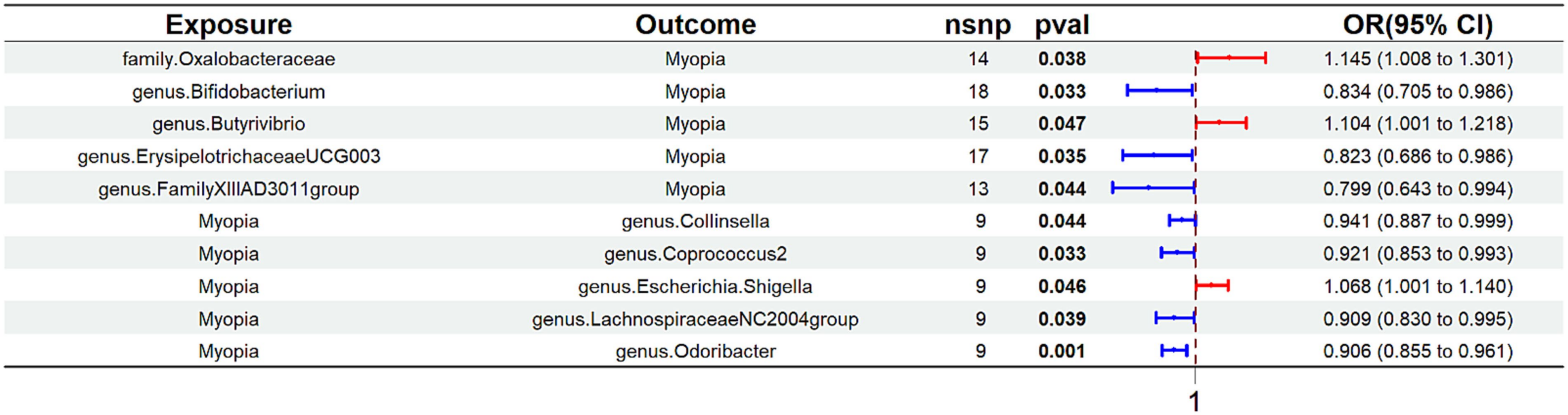
Associations between the GM and myopia risk identified through IVW. IVW, inverse variance weighted.

Through application of the inverse-variance–weighted MR model, we identified causal associations involving five gut bacterial taxa, with two demonstrating positive relationships with increased myopia susceptibility: family Oxalobacteraceae (odds ratio [OR] = 1.145, 95% confidence interval [CI] 1.008–1.301, *p* < 0.05) and genus *Butyrivibrio* (OR = 1.104, 95% CI = 1.001–1.218, *p* < 0.05). Conversely, genus *Bifidobacterium* (OR = 0.834, 95% CI = 0.705–0.986, *p* < 0.05), genus *Erysipelotrichaceae* UCG003 (OR = 0.823, 95% CI = 0.686–0.986, *p* < 0.05), and genus FamilyXIIIAD3011group (OR = 0.799, 95% CI = 0.643–0.994, *p* < 0.05) demonstrated protective effects against myopia (Figure 2; Supplementary Tables S3, S4).

Inverse MR analysis examining the relationship between myopia and GM species revealed that myopia onset was associated with reduced abundance of genus *Collinsella* (OR = 0.941, 95% CI = 0.887–0.999, *p* < 0.05), genus *Coprococcus* 2 (OR = 0.921, 95% CI = 0.853–0.993, *p* < 0.05), genus *Lachnospiraceae* NC2004 group (OR = 0.909, 95% CI = 0.830–0.995, *p* < 0.05), and genus *Odoribacter* (OR = 0.906, 95% CI = 0.855–0.961, *p* < 0.05). Myopia onset was associated with increased abundance of genus *Escherichia*/*Shigella* (OR = 1.068, 95% CI = 1.001–1.140, *p* < 0.05; Figure 2; Supplementary Tables S5, S6).

### 3.2 Sensitivity analysis

To strengthen the inferred causal relationships between the GM and myopia, sensitivity analyses were performed, including a leave-one-out analysis, Q-tests, and Egger’s intercept evaluation. The results indicated no significant heterogeneity or directional horizontal pleiotropy across the IVW and MR-Egger models (*p* > 0.05; Supplementary Tables S7–S10; Supplementary Figures S1–S10).

### 3.3 GM in animal experiments profiles

Table 1 illustrates the changes in SE and AL between groups before and after modeling. At baseline, no statistically significant differences existed in SE or AL between groups (all *p* > 0.05). However, following 4 weeks of form deprivation, the FDM group exhibited a pronounced myopic shift in both refraction (FDM: −4.50 ± 0.43D vs. NC: −0.31 ± 1.23D, *p* < 0.0001) and AL (FDM: 8.52 ± 0.04 mm vs. NC: 8.25 ± 0.09 mm, *p* < 0.0001) compared to values in the NC group.

**Table 1:** Eyeball parameters of guinea pigs before and after experimental induction.

Group	0 week		2 week		4 week	
	AL (mm)	SE (D)	AL (mm)	SE (D)	AL (mm)	SE (D)
NC (*n* = 9)	7.88 ± 0.07	3.44 ± 0.67	8.18 ± 0.09	0.25 ± 1.61	8.25 ± 0.09	−0.31 ± 1.23
FDM (*n* = 9)	7.95 ± 0.08	3.06 ± 0.57	8.33 ± 0.07	−2.83 ± 0.70	8.52 ± 0.04	−4.50 ± 0.43
*p*	0.0815	0.2035	0.0009	0.0001	<0.0001	<0.0001

**Figure 3:**
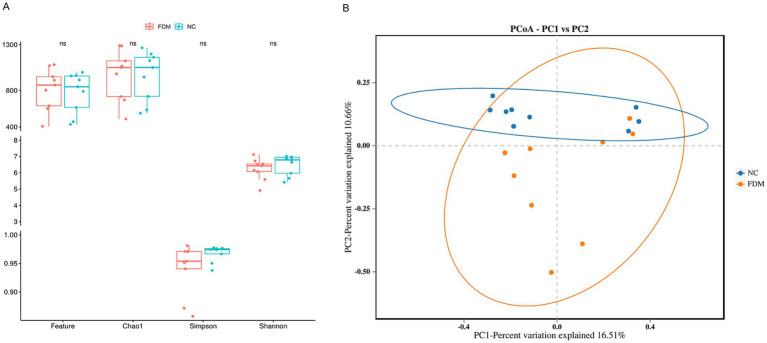
**(A)** Differences in alpha diversity between NC and FDM based on the ob-served Feature, Chao1, Shannon, and Simpson indices. **(B)** Beta diversity differences between the NC and FDM were estimated by Principle coordinates analysis (PCoA).

Following data filtration, we obtained 834,367 high-quality sequences across 18 samples, identifying 14,283 OTUs. Rarefaction curves for richness and diversity metrics plateaued for both sample groups, confirming adequate sequencing depth. Alpha diversity analysis revealed no significant differences between the NC and FDM groups across the Feature, Chao, Shannon, and Simpson indices (Figure 3A). However, PERMANOVA analysis based on Bray–Curtis distance matrices demonstrated significant *β*-diversity differences between the microbial communities of the two groups (*p* < 0.05; Figure 3B).

### 3.4 Myopia-related changes in the composition of gut microflora

Taxonomic dependency analysis revealed that both NC and FDM groups harbored 13 phyla, with Firmicutes, Bacteroidota, and Verrucomicrobiota representing the most abundant taxa. Firmicutes dominated both groups, comprising 49.75 and 46.04% of the GM in the FDM and NC groups, respectively. The FDM group exhibited a higher proportion of Bacteroidota compared to the NC group (34.07% vs. 31.82%; Supplementary Figure S11A). Using the Wilcoxon rank-sum test (Mann–Whitney U test), we identified significant differences in the relative abundances of 26 genera between the NC and FDM groups. Supplementary Figure S11B displays the relative abundance heat map.

**Figure 4:**
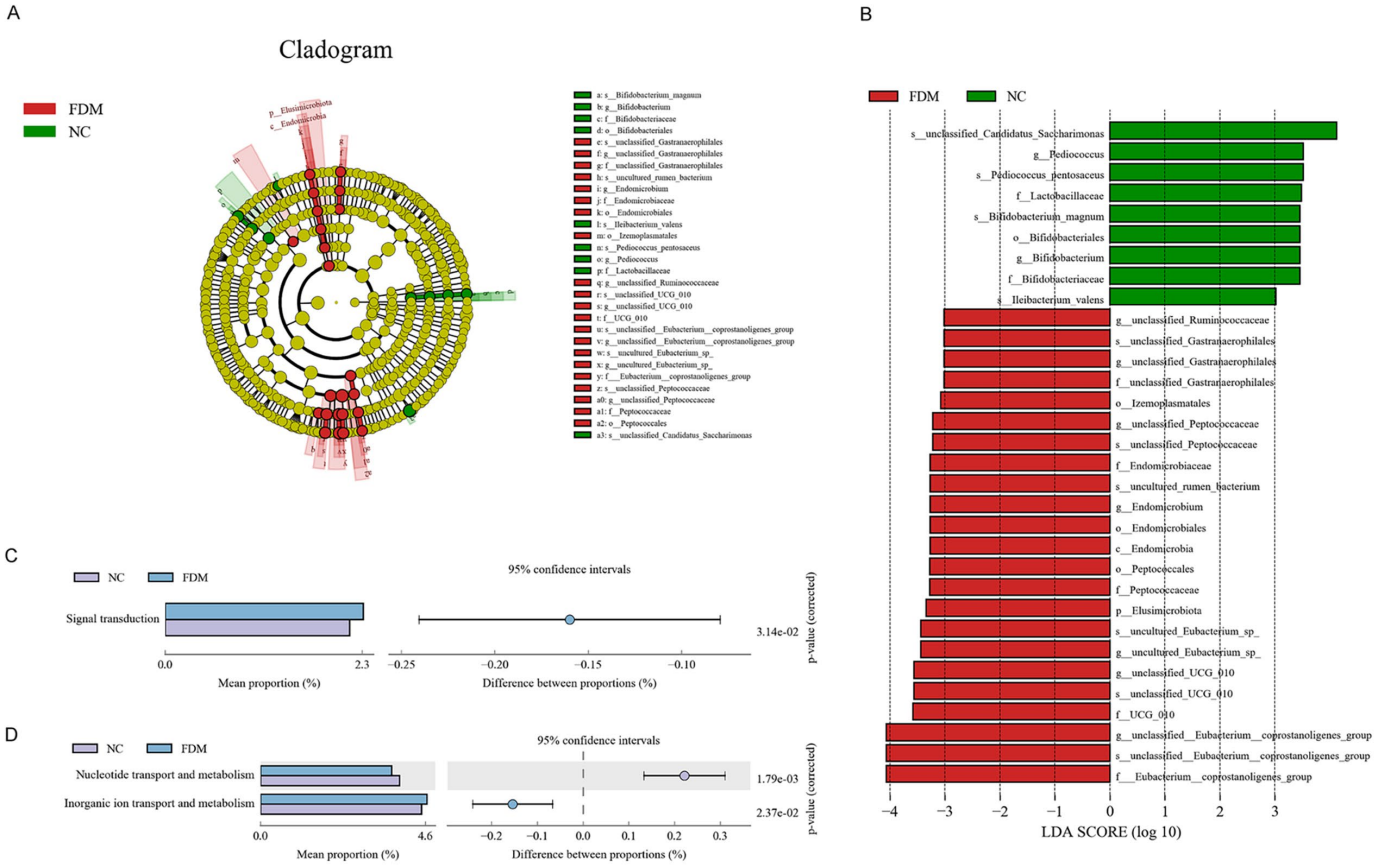
Gut‑microbiota composition and predicted functions in form‑deprivation myopia (FDM) and normal control (NC) guinea pigs. **(A)** Relative abundance of bacterial phyla in the FDM and NC groups. **(B)** Heatmap displaying the 26 operational taxonomic units (OTUs) that differed significantly between groups. **(C)** Predicted KEGG functions: box plot showing enrichment of signal‑transduction pathways in FDM. **(D)** Predicted COG categories: box plots showing decreased nucleoside transport and metabolism and increased inorganic‑ion transport and metabolism in FDM.

To elucidate the differences in taxonomic abundance between the FDM and NC groups, we generated a cladogram using the LEfSe methodology (Figure 4A). This analysis revealed 32 OTUs with significant differences (LDA score>3). Unclassified *Candidatus* Saccharimonas and *Pediococcus pentosaceus* demonstrated higher abundance in the NC group compared to the FDM group. Conversely, the *Eubacterium coprostanoligenes* group and unclassified *Eubacterium coprostanoligenes* group exhibited significantly elevated abundance in the FDM group relative to the NC group (Figure 4B). Importantly, genus *Bifidobacterium* and species *Ileibacterium valens* showed significant enrichment in the NC group (LDA score>3).

### 3.5 Microbial network structure and functional differences

Network analysis unveiled distinct structural differences between NC and FDM groups, despite equivalent node and edge numbers in both networks. The NC group demonstrated superior modularity (0.6396 vs. 0.5327), clustering coefficient (0.467 vs. 0.406), and betweenness centralization (0.259 vs. 0.140). Conversely, the FDM group displayed reduced average path length and increased network diameter (Supplementary Figures S12, S13).

Predictive functional analysis revealed pronounced functional differences between the NC and FDM groups. KEGG analysis demonstrated significant enrichment of differentially expressed GM in signal transduction pathways (*p*-adjusted = 0.0314), indicating upregulation of these pathways under myopic conditions (Figure 4C). COG analysis revealed significant reduction in nucleoside transport and metabolism within the FDM group (*p* = 0.0018), while inorganic ion transport and metabolism showed significant elevation (*p* = 0.024; Figure 4D). Although the NC group exhibited marginally higher relative frequency of translation, ribosomal structure, and biogenesis, these differences lacked statistical significance (*p* = 0.123).

## 4 Discussion

This investigation represents the first application of bidirectional two-sample MR combined with 16S rRNA sequencing to examine the potential relationship between GM and myopia risk. We specifically investigated whether particular gut microbial taxa might modulate myopia risk, thereby advancing understanding of the gut-ocular axis. Our MR analysis revealed that family Oxalobacteraceae and genus *Butyrivibrio* correlated positively with increased myopia risk, while genus *Bifidobacterium*, genus *Erysipelotrichaceae* UCG003, and genus Family XIII AD3011 exhibited protective effects against myopia. Inverse MR findings demonstrated associations between myopia onset and decreased abundance of specific bacterial species, including genus *Collinsella*, genus *Coprococcus* 2, genus *Lachnospiraceae* NC2004 group, and genus *Odoribacter*. Additionally, our analysis identified increased genus *Escherichia*/*Shigella* abundance associated with myopia onset. Full-length 16S sequencing of guinea pig fecal samples revealed significant genus *Bifidobacterium* and species *Ileibacterium valens* enrichment in the NC group, corroborating our MR findings.

Both MR analysis and 16S rRNA sequencing substantiated the association between *Bifidobacterium* and decreased myopia risk. While the ORs are small (OR = 0.834), our instruments’ high F- statistics imply that weak bias is unlikely (33). Prior investigations have suggest that *Bifidobacterium* can ameliorate ocular diseases through inhibition of oxidative stress-induced inflammatory responses (34, 35). *Bifidobacterium* administration to mice with compromised retinal ganglion cells reduced microglial number and activation while enhancing Müller cell activation. This process coincided with inflammatory cytokine downregulation and neurotrophic factor upregulation (35). As a probiotic organism, *Bifidobacterium* generates short-chain fatty acids (SCFAs), including acetic acid (36). SCFAs have been reported to modulate inflammatory factor expression (e.g., tumor necrosis factor tumor necrosis factor (TNF)-*α*, interleukin interleukin (IL)-6, IL-10) through G protein-coupled receptor activation and other receptor pathways (37, 38) or via histone deacetylase inhibition (39). One investigation revealed that proinflammatory cytokines IL-6 and TNF-α synergistically facilitate axial elongation within the inflammatory microenvironment characteristic of myopic eyes (40). IL-6 triggers proinflammatory signaling through soluble IL-6 receptor binding and subsequent JAK/MAPK and NF-κB pathway activation, resulting in oxidative stress, endothelial dysfunction, and retinal inflammation (41–44). Thus, we speculate that SCFAs produced by Bifidobacterium could help down regulate inflammatory factors such as IL-6 and TNF-α, potentially contributing to slower myopic axial elongation. We note, however, that this mechanism remains hypothetical and was not directly shown by our data. Furthermore, *Bifidobacterium* demonstrates substantial antioxidant capabilities. Reactive oxygen species (ROS), primary mediators of oxidative stress, can be induced by mechanical stretching (45), a process intimately associated with AL elongation during myopia progression (46, 47). Zeng et al. (48) demonstrated that *Bifidobacterium* NCU-08 attenuated neuronal apoptosis and ROS generation through PI3K/AKT signaling pathway upregulation, consequently enhancing antioxidant enzyme activities, including glutathione peroxidase, superoxide dismutase, and catalase.

Recent investigations have yielded contradictory findings regarding *Bifidobacterium*’s role in myopia. Li et al. (14) documented enrichment in myopic mice, while Omar et al. (16) reported elevated abundance in myopic humans. These inconsistencies likely stem from species-specific variations in microbial ecology, including functional divergence among murine, human, and guinea pig models, as well as methodological constraints. Cross-sectional human studies cannot adequately control for potential confounding variables, particularly dietary factors. In contrast, our MR analysis combined with animal experimentation, provides compelling evidence supporting *Bifidobacterium*’s causal protective role. Crucially, *Bifidobacterium* encompasses diverse subspecies exhibiting strain-specific effects. Protective strains identified in guinea pig models may differ from those present in human myopes. Future investigations should emphasize strain-level characterization within human cohorts and examine probiotic interventions to elucidate context-dependent effects.

Our MR analysis identified *Erysipelotrichaceae* UCG003, a genus within the Erysipelotrichaceae family, as a protective factor against myopia. Animal experiments revealed significant enrichment of *Ileibacterium valens*, another Erysipelotrichaceae family member, in the NC group. Erysipelotrichaceae, classified within the phylum Firmicutes, comprise Gram-positive, slender, filamentous, rod-shaped bacteria (49). Erysipelotrichaceae abundance varies considerably across hosts, demonstrating elevation in inflammatory mouse models but reduction in human inflammatory bowel disease (IBD) patients (50, 51). Palm et al. (52) found no significant abundance differences for specific immunogenic Erysipelotrichaceae species between IBD patients and healthy controls; however, subsequent germ-free mouse infection experiments demonstrated that colonization with IgA-coated Erysipelotrichaceae-containing bacterial communities induced more severe colitis responses. Zhao *et al.* documented increased *Erysipelotricaceae incertae sedis* levels in mice receiving high-dose gentamicin treatment (53). These findings suggest that *Erysipelotrichaceae* play a crucial role in intestinal immune defense, with immunoregulatory properties that could be protective in reducing ocular inflammation, which is a key factor in the progression of myopia. Nonetheless, given the preliminary nature of the current research on *Erysipelotrichaceae*, these results should be interpreted with caution. Future studies should investigate the specific roles of different genera and species within the *Erysipelotrichaceae* family to clarify their distinct effects on myopia and validate our findings.

The NC group’s microbial network displayed enhanced modularity and cohesion, suggesting a more stable microecological environment potentially better suited to maintaining host-microbe equilibrium. KEGG analysis demonstrated significant GM enrichment in signal transduction pathways. Additionally, COG analysis revealed marked nucleoside transport and metabolism reduction coupled with substantially elevated inorganic ion transport and metabolism in the FDM group. Previous investigations have established the retina’s substantial energy demands (54), and decreased nucleoside transport and metabolism may restrict retinal cell energy supply. Ganglion cells demonstrate particular vulnerability to energy deficiency due to their exceptionally active metabolism (55). Our study observed an increase in inorganic ion transport in the FDM group. Wu et al. (56) similarly documented potassium flux alterations in ciliary muscles of guinea pigs with lens-induced myopia, identifying myopia-induced potassium influx. Additionally, Zhong et al. (57) reported increased Piezo1 expression—a mechanically sensitive ion channel—in FDM guinea pig retinas. Notably, Piezo1 channel function inhibition slows myopia progression and partially reduces retinal ROS levels in FDM guinea pigs. Collectively, these findings suggest that enhanced inorganic ion transport, particularly through mechanisms including inward potassium flux and Piezo1 ion channels, may constitute a critical factor in myopia development. Modulation of these pathways may provide promising therapeutic approaches for addressing retinal oxidative stress and decelerating myopia progression.

Unlike previous investigations, our study employed MR analysis to effectively eliminate confounding factors and reverse causation bias. This approach circumvents limitations inherent in traditional randomized controlled trials, including ethical constraints, extended observation periods, and substantial costs. Our findings underwent rigorous robustness checks to enhance their credibility. Distinguishing our work from previous MR studies, we utilized MR as an exploratory tool for identifying GM potentially influencing myopia, subsequently validating these findings through animal experimentation to ensure comprehensive and reliable results.

Despite employing rigorous analytical methods, the inherent observational nature of Mendelian randomization (MR) and microbiome sequencing studies means residual confounding factors—such as dietary patterns, antibiotic use and lifestyle behaviors—cannot be fully excluded (58, 59). Moreover, both gut-microbiome composition and the distribution of myopia risk factors vary markedly across ethnic and geographic groups, limiting the external validity of our findings beyond the predominantly European ancestry examined. Our microbiome analysis predominantly identifies Bifidobacterium at the genus level, along with a limited number of abundant species. This approach does not allow for the capture of fine-scale strain differentiation or the functional gene content of the microbiota. Given that Bifidobacterium is a heterogeneous genus consisting of numerous species with overlapping 16S rRNA gene sequences but distinct metabolic functions, our findings may not fully reflect the nuanced biological roles of specific strains. Finally, MR estimates represent the lifelong effect of genetically proxied variation. Because myopia typically manifests during childhood and adolescence, the impact of the gut microbiota during critical developmental windows may differ from the average lifetime effect inferred in adult MR analyses. Findings should therefore be interpreted cautiously when extrapolating to age-specific microbiome–host interactions.

*Bifidobacterium* species demonstrate widespread use as oral probiotics with established long-term safety profiles. A clinical trial administering approximately 3 × 10^9^ CFU/day of *B. infantis* or *B. bifidum* to infants for 8 weeks reported no serious adverse events (60). Consequently, *Bifidobacterium*-based interventions are generally well tolerated. When planning myopia prevention trials, researchers must select appropriate strains, doses, and delivery methods to ensure the most effective outcomes. Oral delivery remains most feasible (e.g., capsules or fortified foods). Nevertheless, optimal dosing, duration, and timing (particularly in pediatric populations) require further investigation.

## 5 Conclusion

In summary, our investigation provides evidence that Bifidobacterium might exert protective effects against myopia. These findings suggest that Bifidobacterium supplementation could be a promising strategy for myopia risk reduction. Future research should prioritize the development and evaluation of *Bifidobacterium*-based interventions to establish their efficacy in myopia control.

## Data Availability

The original contributions presented in the study are included in the article/Supplementary material, further inquiries can be directed to the corresponding authors.

## Footnotes

^1^ https://www.drive5.com/usearch/

^2^ http://www.arb-silva.de

^3^ https://qiime2.org/
