# Supplementary material for: Potential protective role of Bifidobacteria in myopia prevention: evidence from full-length 16S rRNA sequencing and bidirectional Mendelian randomization analysis
Source: Front Med (Lausanne). 2025 Aug 13;12:1634120. doi: 10.3389/fmed.2025.1634120 (PMC12380757; doi:10.3389/fmed.2025.1634120)
Supplement: Supplementary file 2 [file Data_Sheet_2.docx]

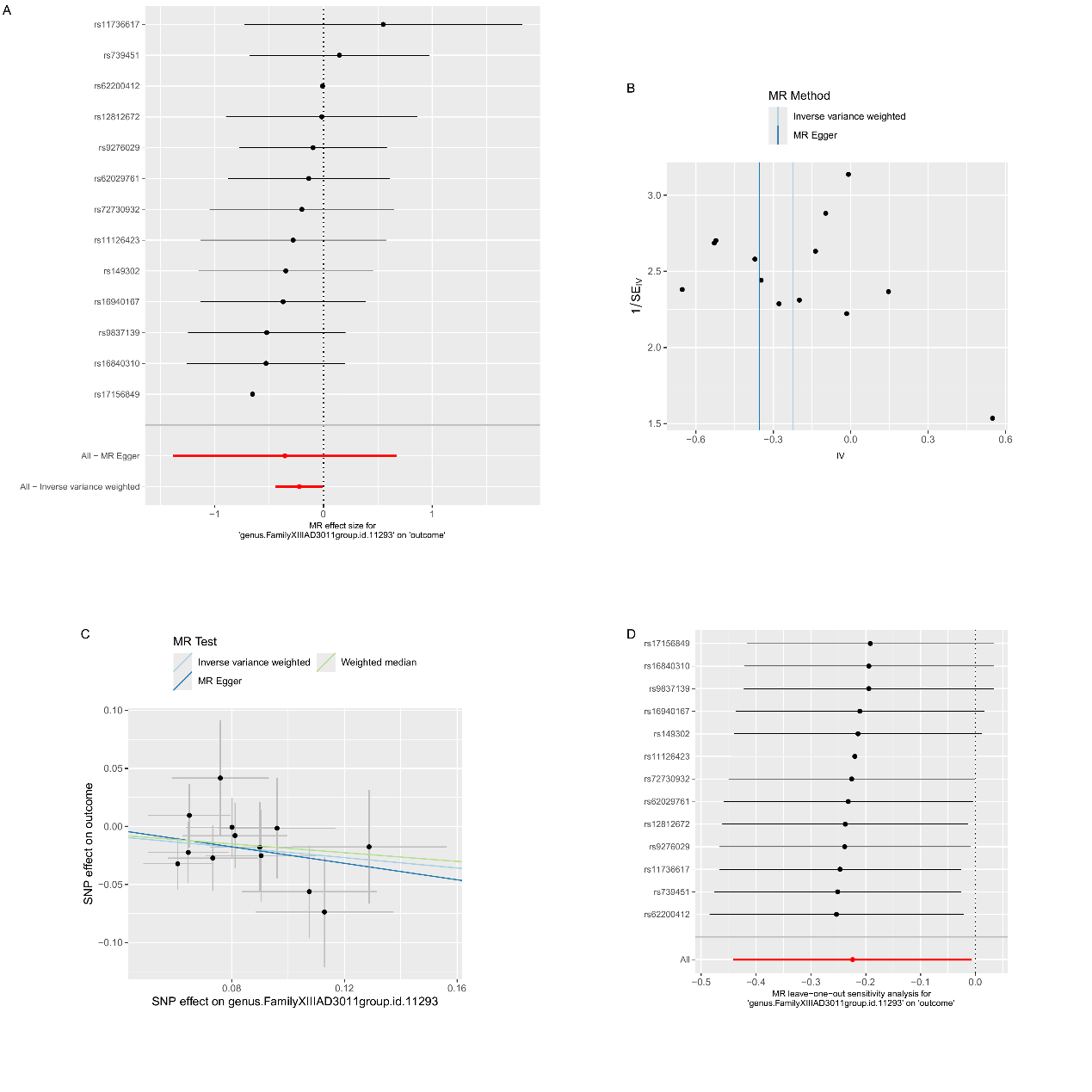


Supplementary Figure 1. Mendelian randomization analyses of effects of genus.FamilyXIIIAD3011group on myopia. A, Forest plot; B, Funnel plot; C, Scatter plot; D, Leave-one-out plot.


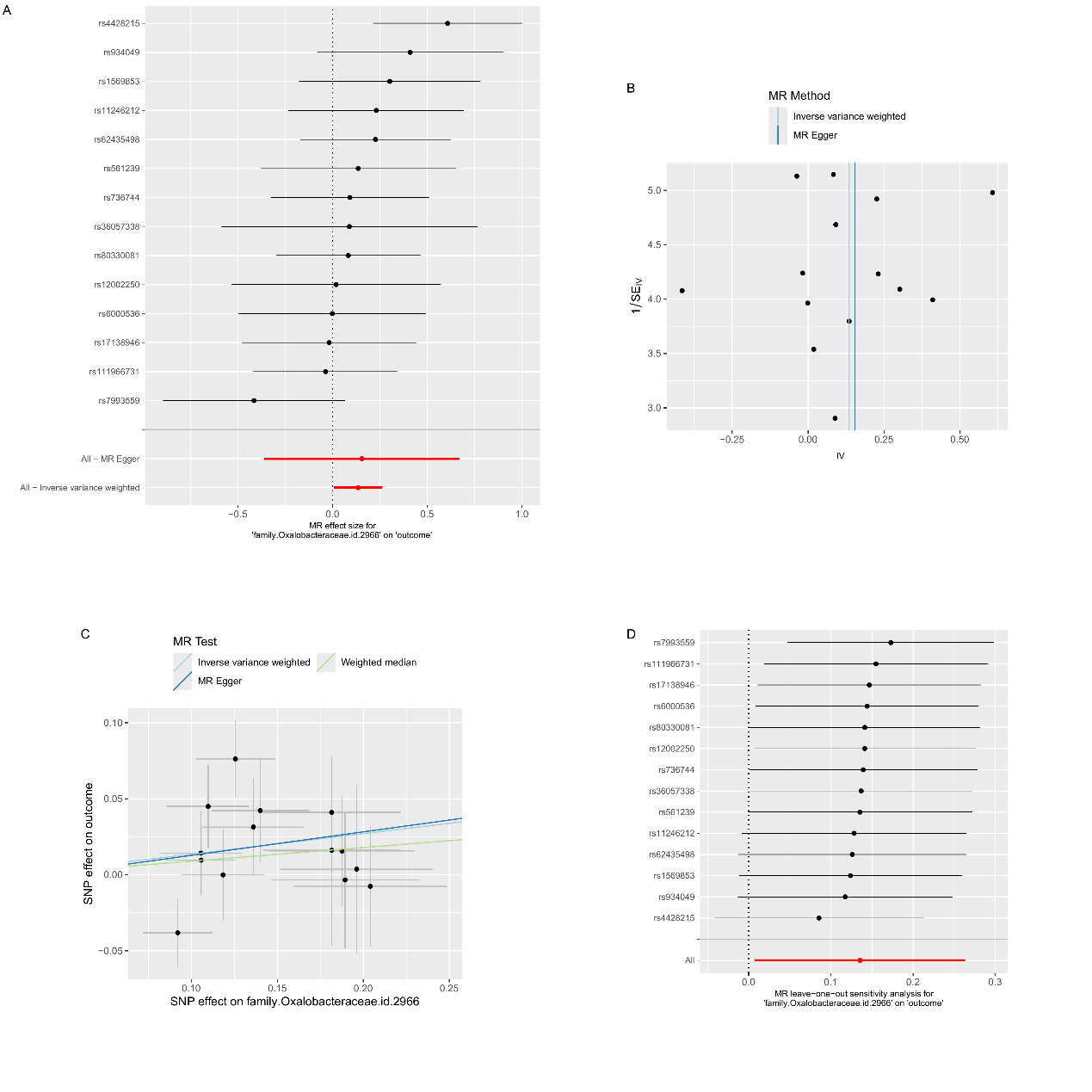


Supplementary Figure 2. Mendelian randomization analyses of effects of family.Oxalobacteraceae on myopia. A, Forest plot; B, Funnel plot; C, Scatter plot; D, Leave-one-out plot.


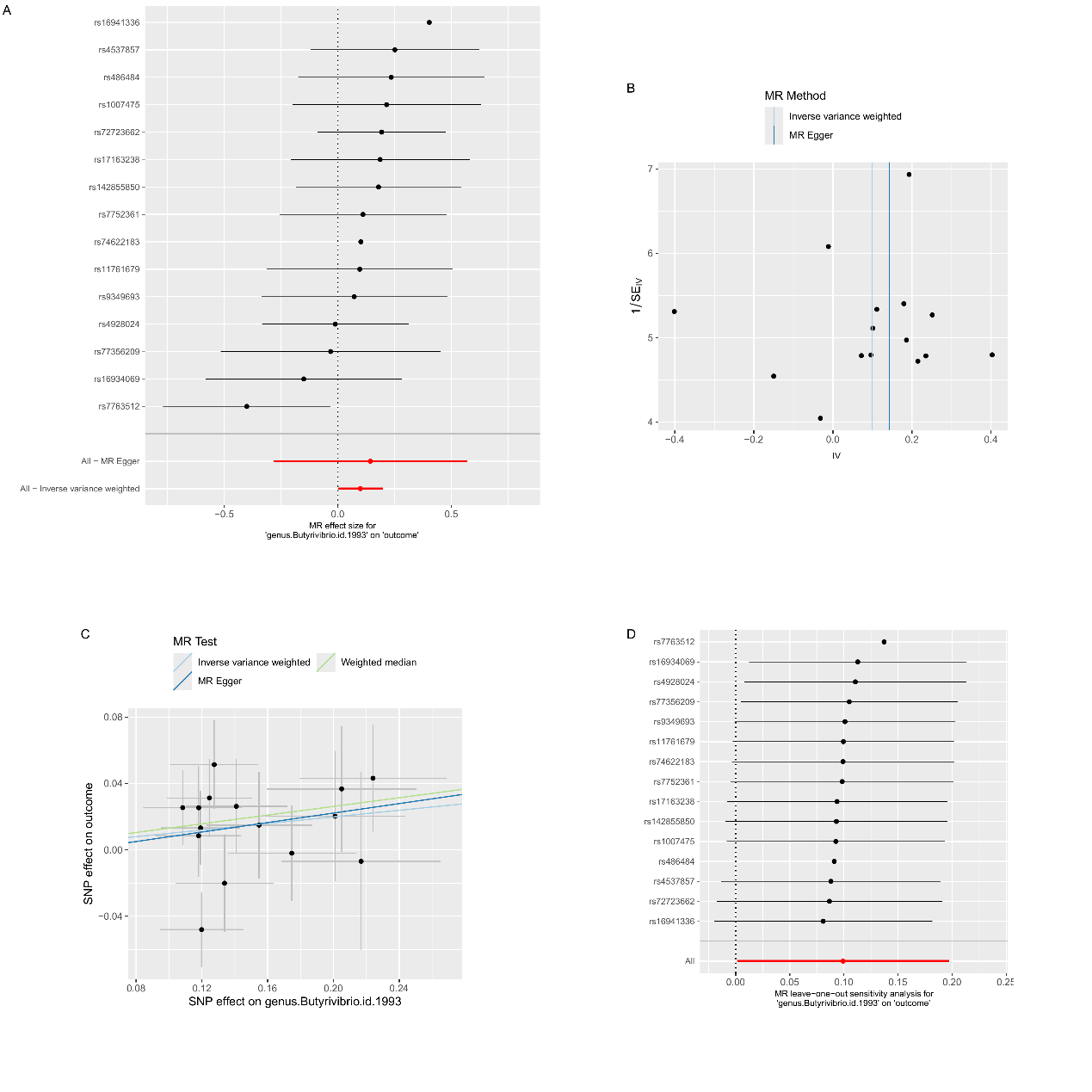


Supplementary Figure 3. Mendelian randomization analyses of effects of genus.Butyrivibrio on myopia. A, Forest plot; B, Funnel plot; C, Scatter plot; D, Leave-one-out plot.


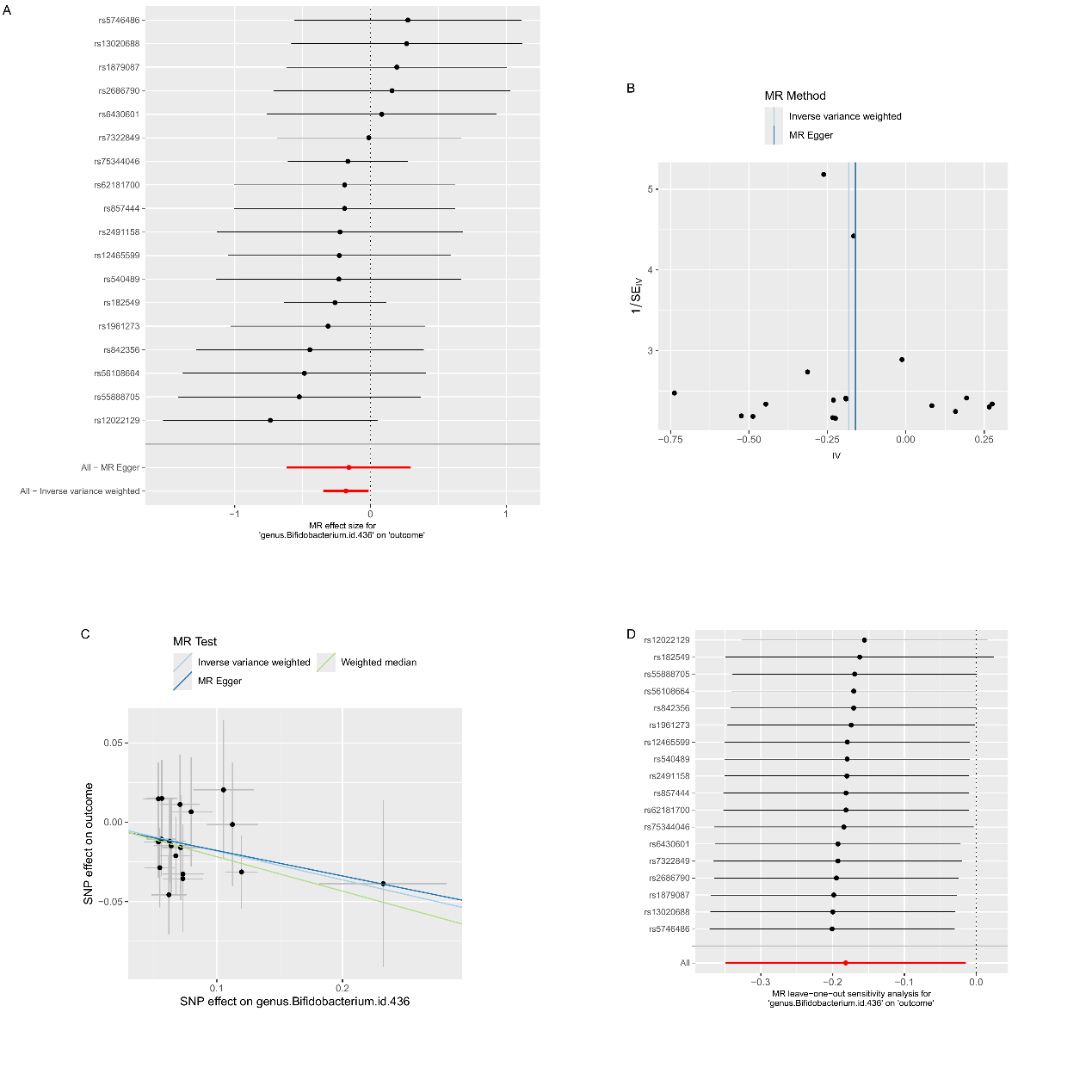


Supplementary Figure 4. Mendelian randomization analyses of effects of genus.Bifidobacterium on myopia. A, Forest plot; B, Funnel plot; C, Scatter plot; D, Leave-one-out plot.


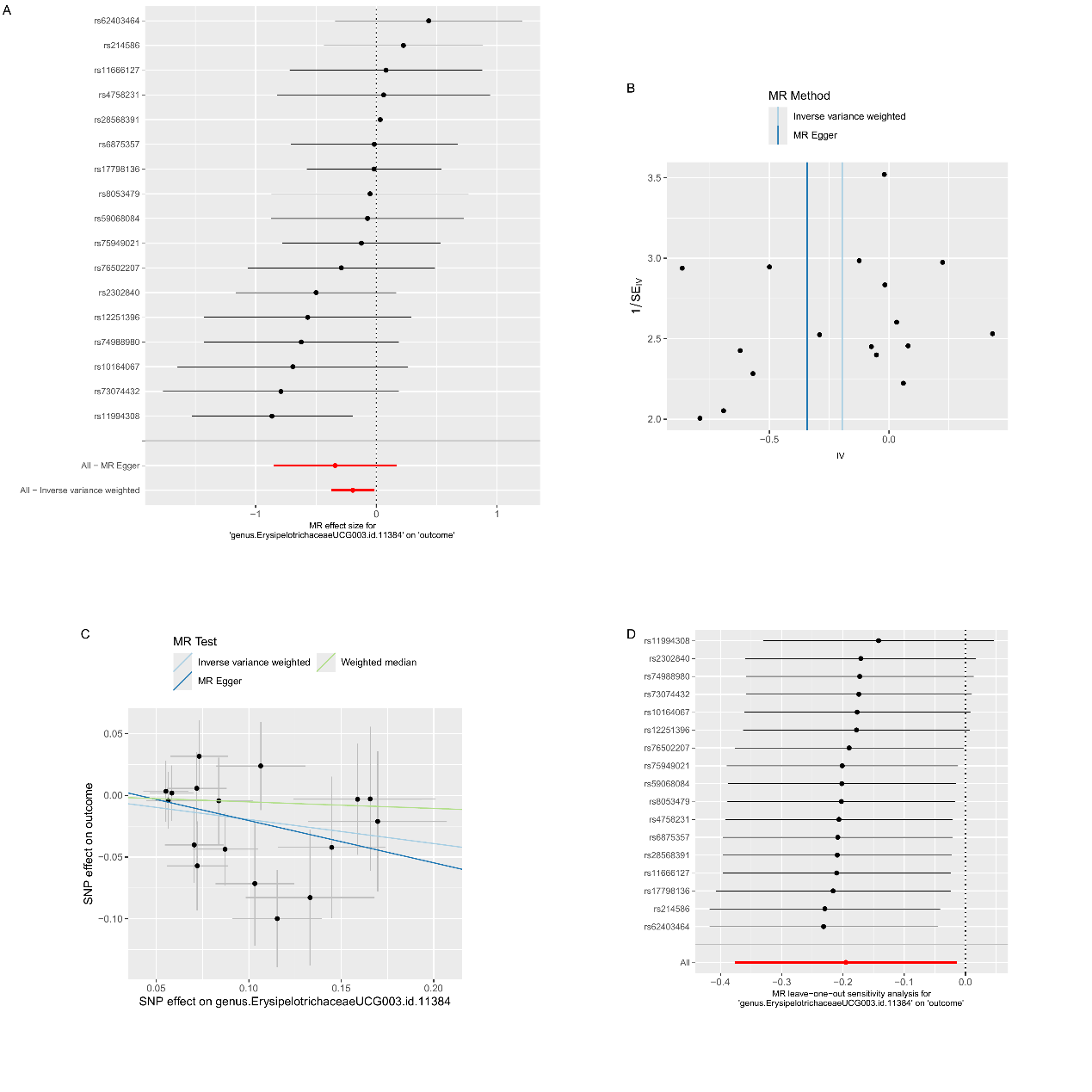


Supplementary Figure 5. Mendelian randomization analyses of effects of genus.ErysipelotrichaceaeUCG003 on myopia. A, Forest plot; B, Funnel plot; C, Scatter plot; D, Leave-one-out plot.


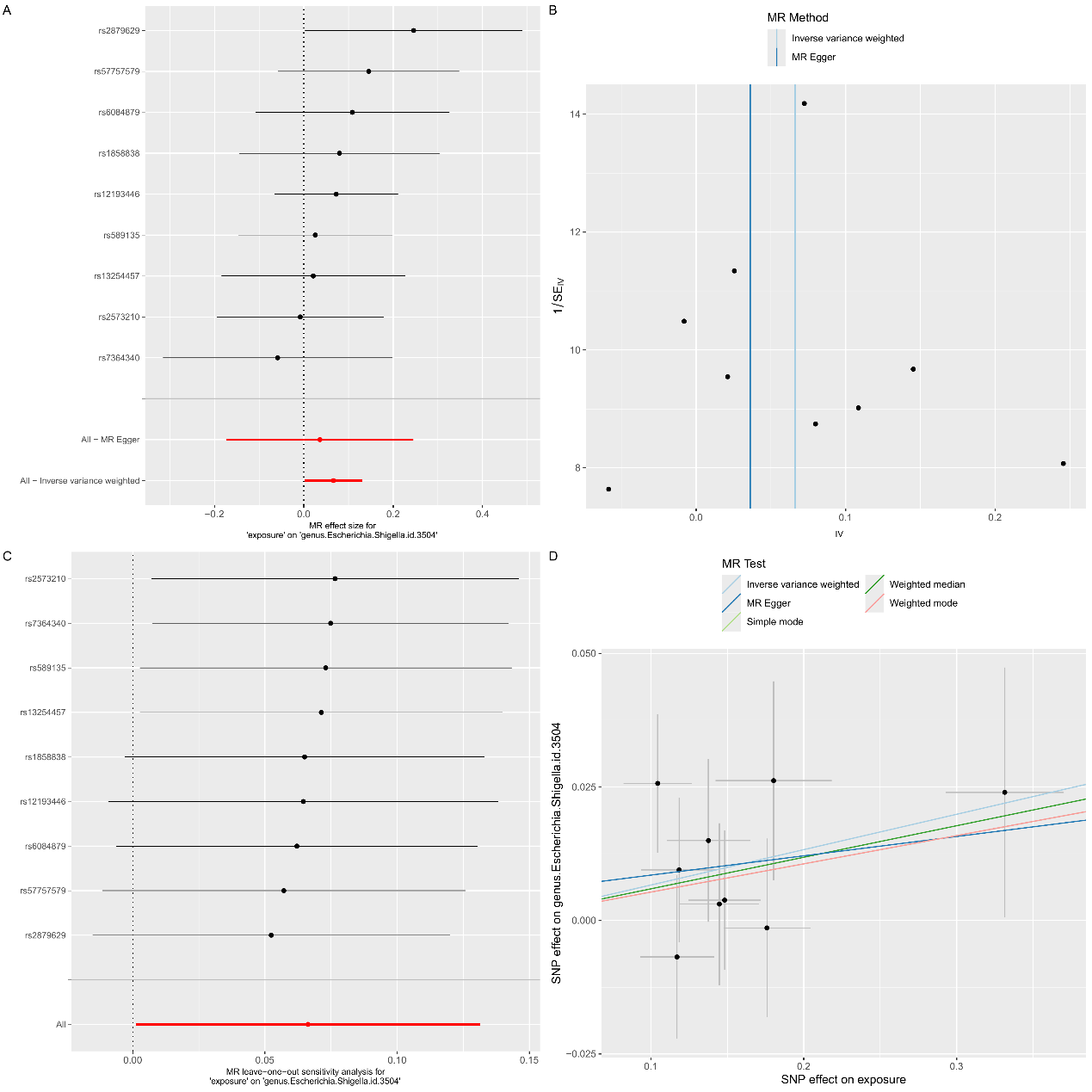

Supplementary Figure 6. Mendelian randomization analyses of effects of myopia on genus.Escherichia.Shigella. A, Forest plot; B, Funnel plot; C, Leave-one-out plot; D, Scatter plot.


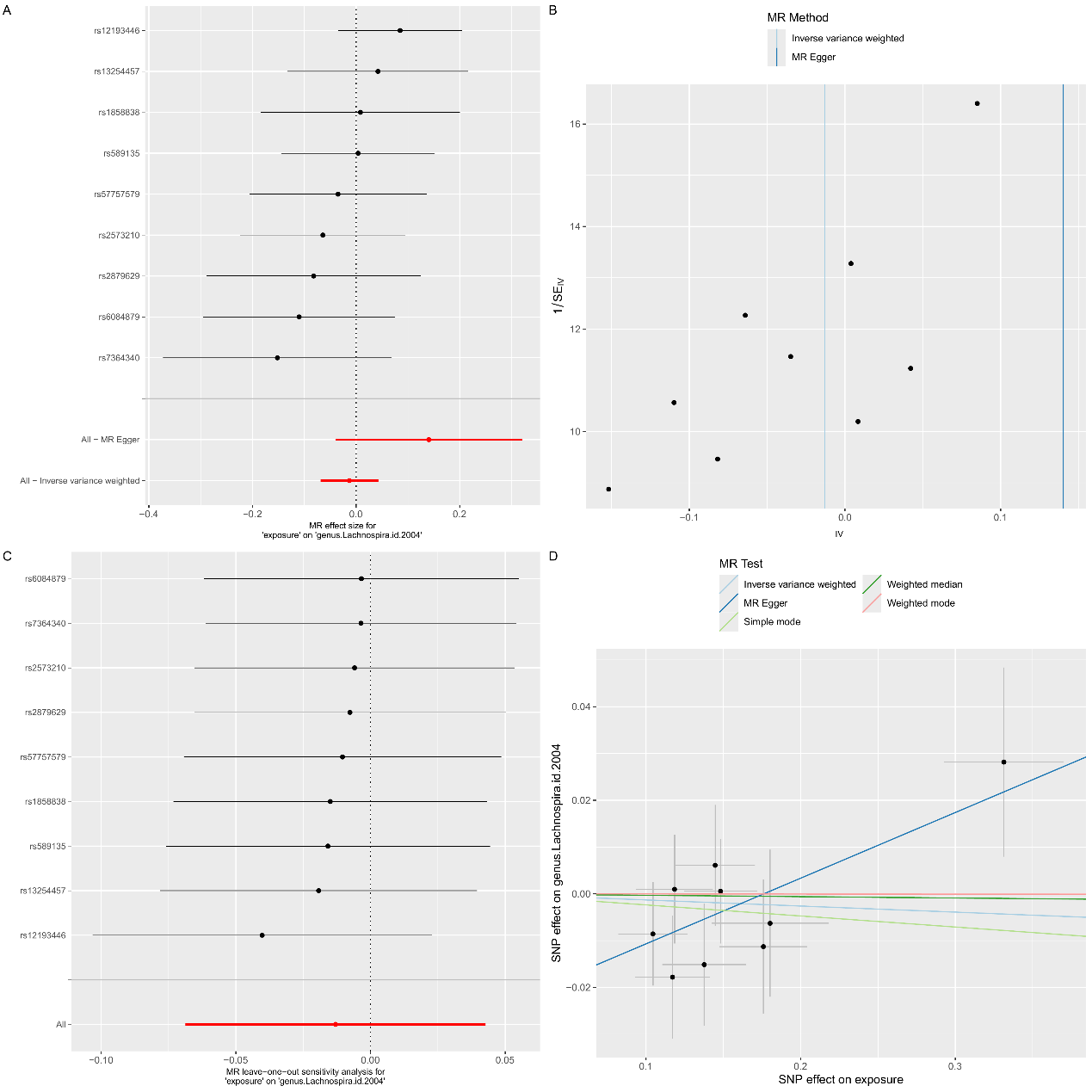


Supplementary Figure 7. Mendelian randomization analyses of effects of myopia on genus.LachnospiraceaeNC2004group. A, Forest plot; B, Funnel plot; C, Leave-one-out plot; D, Scatter plot.


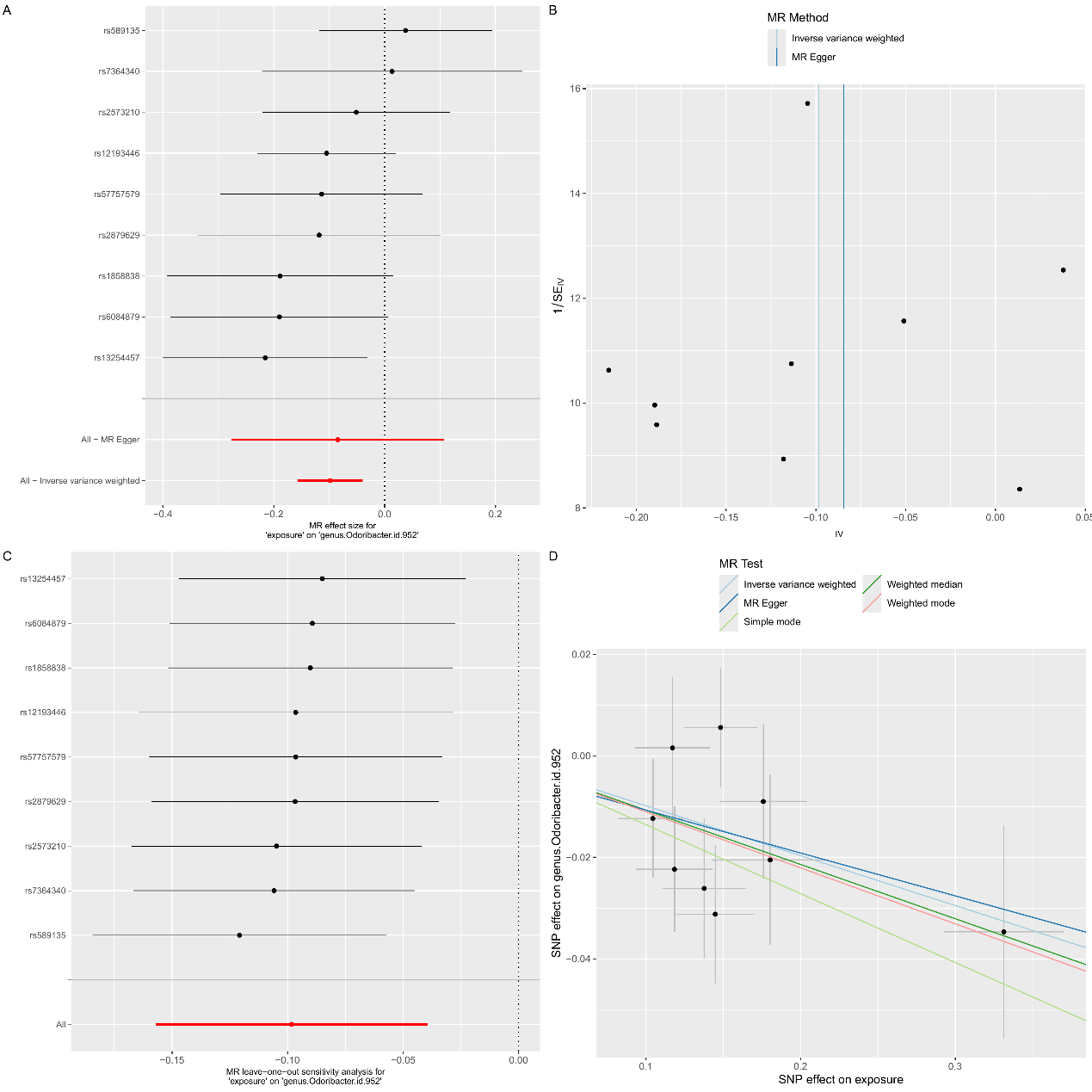


Supplementary Figure 8. Mendelian randomization analyses of effects of myopia on genus.Odoribacter. A, Forest plot; B, Funnel plot; C, Leave-one-out plot; D, Scatter plot.


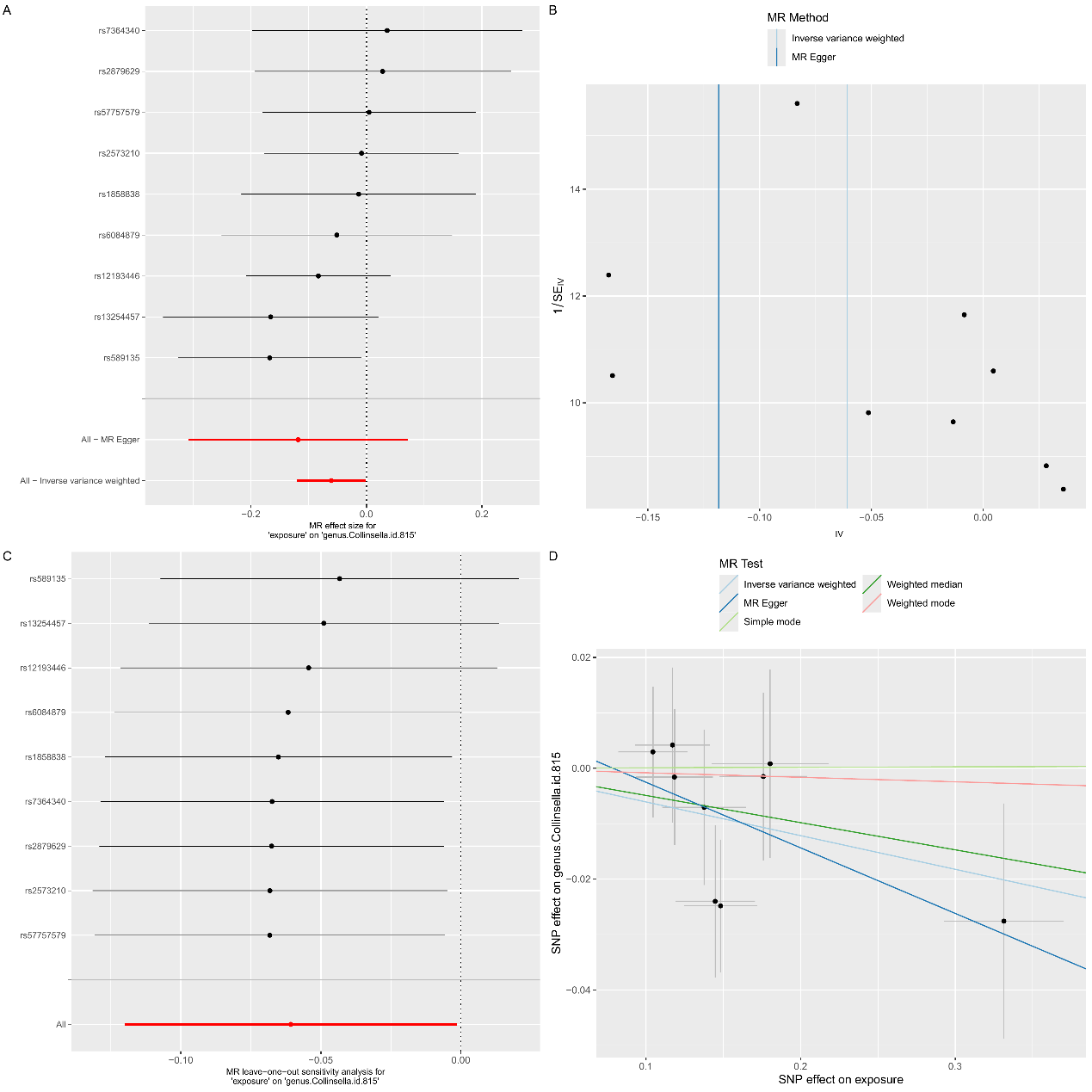


Supplementary Figure 9. Mendelian randomization analyses of effects of myopia on genus.Collinsella. A, Forest plot; B, Funnel plot; C, Leave-one-out plot; D, Scatter plot.


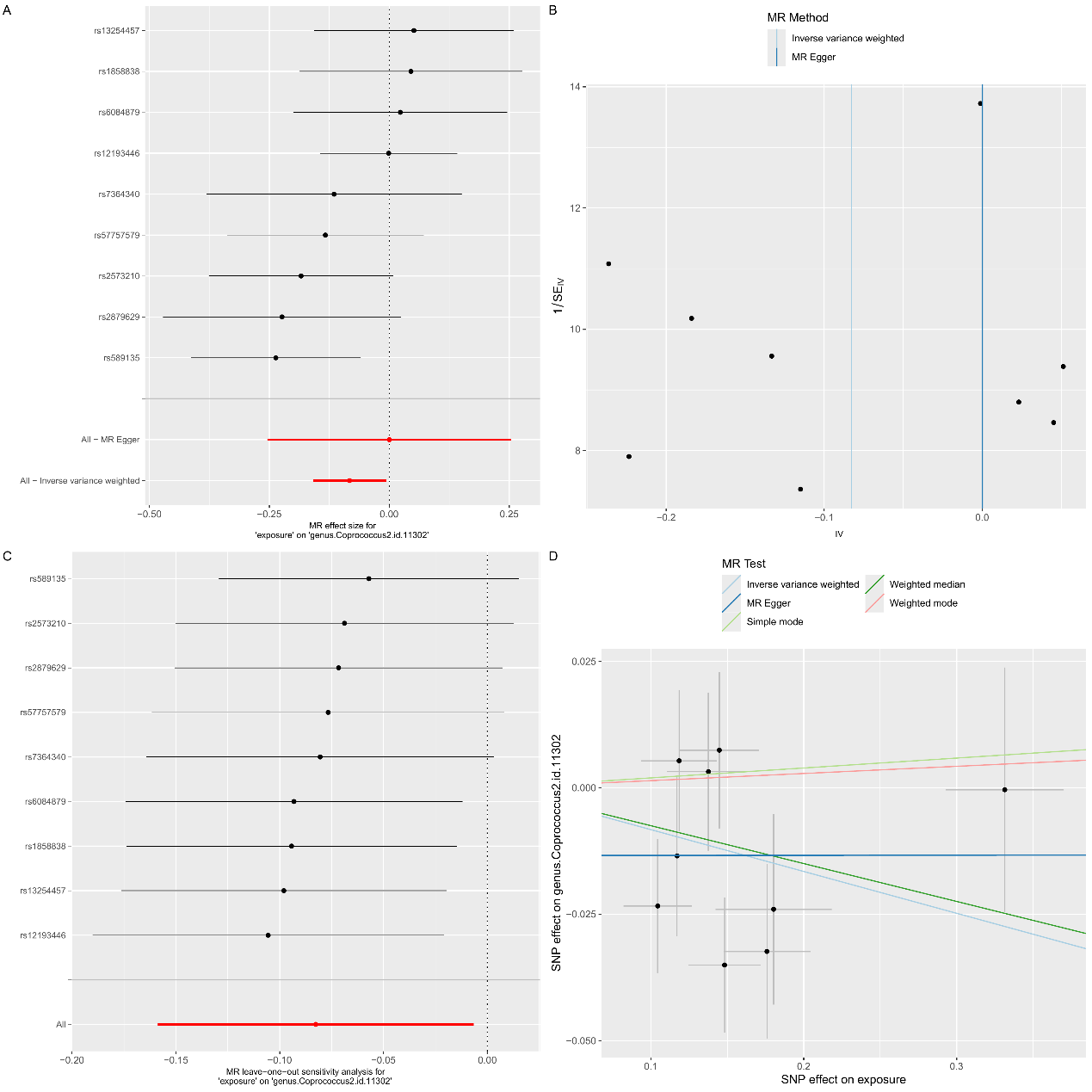


Supplementary Figure 10. Mendelian randomization analyses of effects of myopia on genus.Coprococcus2. A, Forest plot; B, Funnel plot; C, Leave-one-out plot; D, Scatter plot.


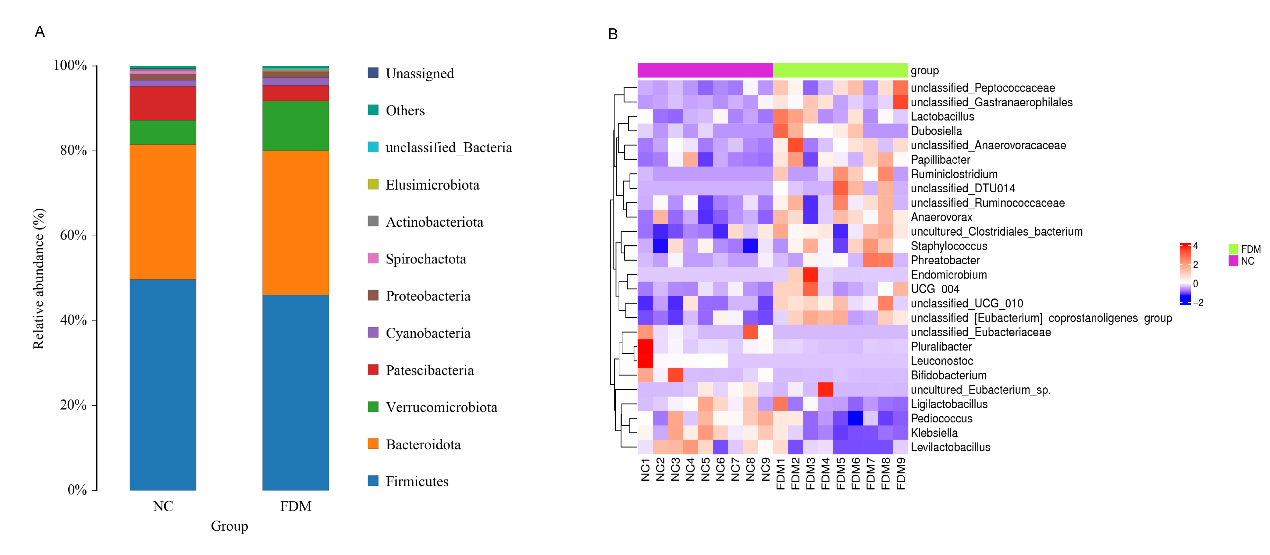


Supplementary Figure 11**.** (A) The proportions at the bacteria phylum level for each group. (B) The heat map displayed the relative abundance of the 26 operational taxonomic units (OTUs) that significantly differed between the FDM and NC groups. OTU data underwent z-transformation, with values ranging from low (blue) to high (red) abundance.


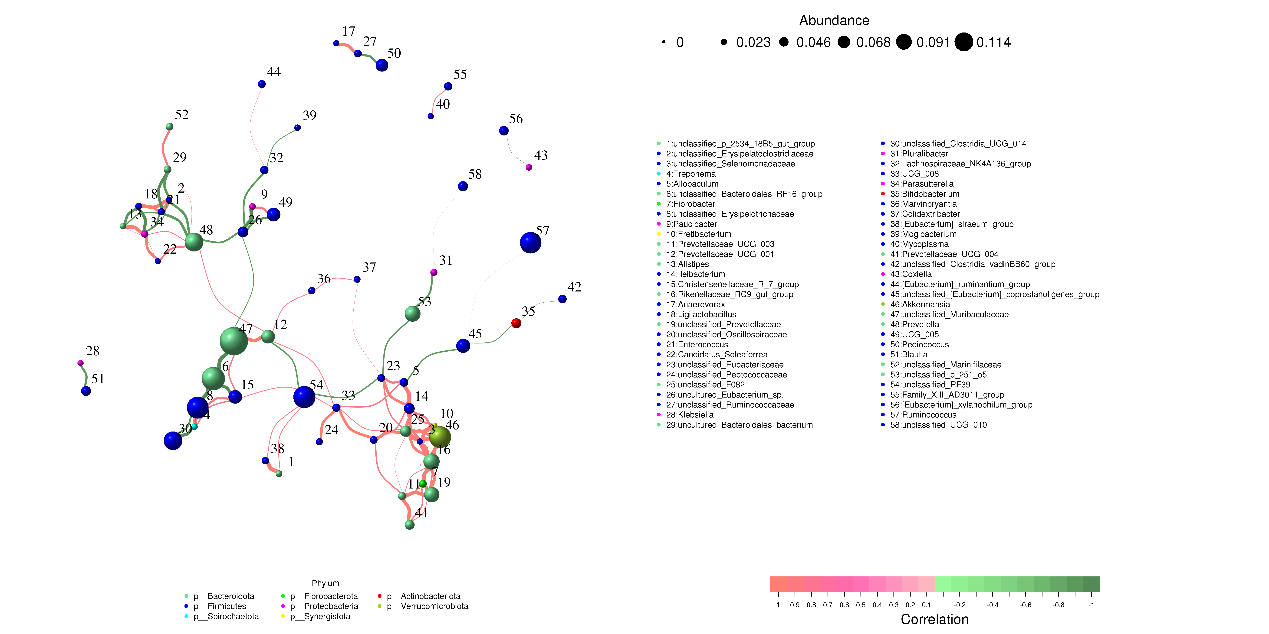


Supplementary Figure 12. Network analysis of co-occurring intestinal microbes in the NC group


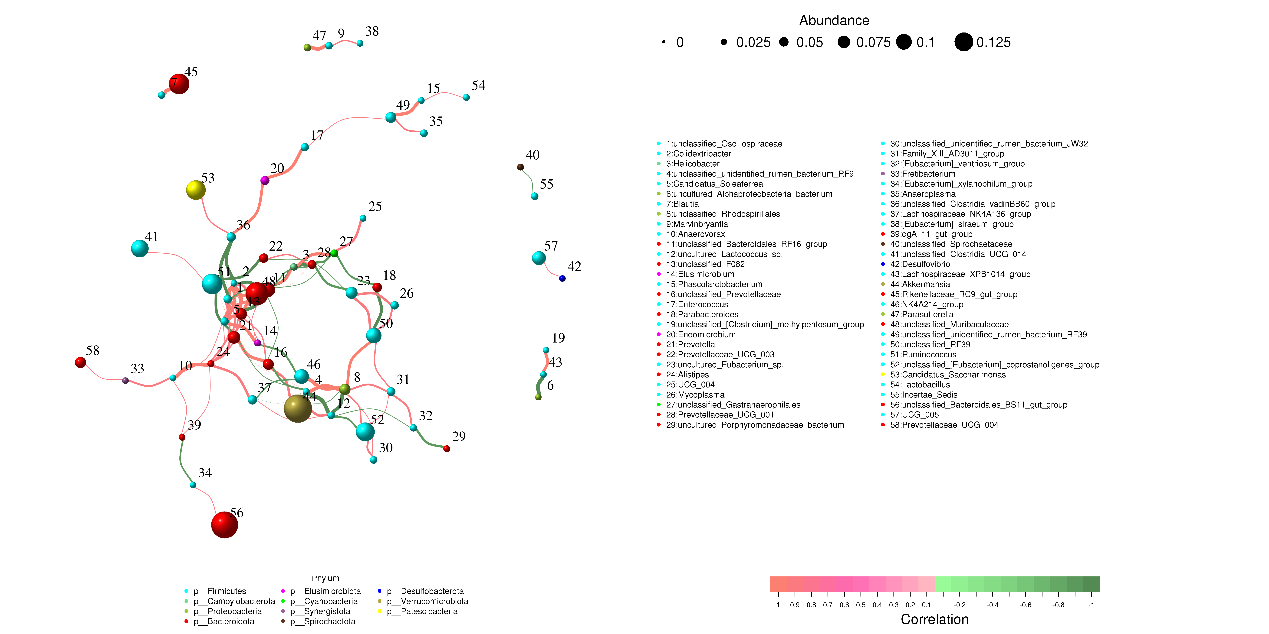


Supplementary Figure 13. Network analysis of co-occurring intestinal microbes in the FDM group
